# Supplementary material for: Development and design of the BELpREG registration system for the collection of real-world data on medication use in pregnancy and mother-infant outcomes
Source: Front Drug Saf Regul. 2023 May 16;3:1166963. doi: 10.3389/fdsfr.2023.1166963 (PMC12443112; doi:10.3389/fdsfr.2023.1166963)
Supplement: Supplementary file 1 [file DataSheet1.pdf]

Supplementary Table and Figure BELpREG

Development and design of the BELpREG registration system for the collection of real-world data on medication use in pregnancy and mother-infant outcomes

Laure Sillis, Veerle Foulon, Karel Allegaert, Annick Bogaerts, Maarten De Vos, Titia Hompes, Anne Smits, Kristel Van Calsteren, Jan Y Verbakel and Michael Ceulemans

Correspondence: Michael Ceulemans (michael.ceulemans@kuleuven.be)

1 Supplementary Table

Overview of the BELpREG data variables, shown per category and indicating in which survey instruments they are asked (i.e., time points at which the information is collected; EM = Pregnancy Enrolment Questionnaire; FU = Pregnancy Follow-up Questionnaire; PP 1 = First Postpartum Questionnaire; PP 2 = Second Postpartum Questionnaire). This overview follows, where possible, the order of the variables as they appear in the BELpREG questionnaires.

**Category 1: Sociodemographics**								
**Variable**	**Definition**	**Values**	**Field type**	**Source**	**Survey Instruments**			
					**EM**	**FU**	**PP 1**	**PP 2**
**1. BELpREG record id**	Unique numeric code	Integer (Automatically assigned)	Text box	Automatically assigned	x	x	x	x
**2. First name**	Participant's first name	Text	Text box	*Derived from eConsent*				
**3. Last name**	Participant's last name	Text	Text box	*Derived from eConsent*				
**4. E-mail address**	E-mail address used for study participation	Validated text structure (e-mail)	Text box	*Derived from eConsent*				
**5. Date of birth**	Date of birth participant	Date (DD-MM-YYYY)	Text box	Reported	x			
**6. Age**	Age of the participant at first day of the LMP[1]	Integer	Calculated field	*Derived*				

**Residence**								
7. Country of residence	Country of current residence	Text - selection from drop-down list[2] or free text	Drop-down list linked to database, Autocomplete	Reported	x			
8. City of residence	Name and postal code of current residence	Text - selection from drop-down list[3] or free text	Drop-down list linked to database, Autocomplete	Reported	x			
**9. Partner**	Currently having a partner	Y/N	Radio buttons	Reported	x			
**10. Father**	Current partner is biological father	Y/N	Radio buttons	Reported	x			
**11. Year of birth biological father**	Year of birth biological father	YYYY	Text box	Reported	x			
**Nationality**								
12. Nationality participant	Nationality participant	Belgian; Dutch; French; German; Moroccan; Romanian; Turkish; Polish; Syrian; Other (specify[2])	Checkboxes, Multiple choice	Reported	x			
13. Nationality biological father	Nationality biological father	Belgian; Dutch; French; German; Moroccan; Romanian; Turkish; Polish; Syrian; Other (specify[2])	Checkboxes, Multiple choice	Reported	x			
**Ethnicity**								
14. Ethnicity participant	Ethnicity of the participant	European; Maghreb; Rest of Africa; Near/Middle East; Pacific/Far East; Rest of Asia; North America; Central and South America; I don't know / I prefer not to say	Checkboxes, Multiple choice	Reported	x			
15. Ethnicity biological father	Ethnicity of the biological father	European; Maghreb; Rest of Africa; Near/Middle East; Pacific/Far East; Rest of Asia; North America; Central and South America; I don’t know / I prefer not to say	Checkboxes, Multiple choice	Reported	x			
**16. Home language**	Language spoken at home	Dutch; French; English; German; Arabic; Turkish; Berber; Other (specify)	Checkboxes, Multiple choice	Reported	x			
**Education**								
17. Highest educational level of participant	Highest educational degree obtained by participant	Primary education; Lower secondary education (certificate 2nd degree); Higher secondary education; Higher vocational education (HBO5); Professional Bachelor / Higher education short type (non-	Radio buttons	Reported	x			

		university); Academic Bachelor; Master; PhD; Other (specify)						
18. Education in healthcare of participant	A diploma in healthcare obtained by the participant	Y; N; Currently following education in healthcare	Radio buttons	Reported	x			
19. Education in healthcare of participant – type	Type of education in healthcare by the participant	Text	Text box		x			
20. Highest educational level of partner	Highest educational degree obtained by current partner	Primary education; Lower secondary education (certificate 2nd degree); Higher secondary education; Higher vocational education (HBO5); Professional Bachelor / Higher education short type (non-university); Academic Bachelor; Master; PhD; Other (specify)	Radio buttons	Reported	x			
**Profession**								
21. Professional activity participant	The participant being professional active in the past year	Y/N	Radio buttons	Reported	x			
22. Professional activity participant - week hours	Number of hours per week participant was professionally active in the past year	< 20h/week; 20-40h/week; 40-60h/week; >60h/week	Radio buttons	Reported	x			
23. Professional activity participant - healthcare	The participant being professional active as healthcare professional in the past year	Y/N	Radio buttons	Reported	x			
24. Occupational exposure to substances participant	Occupational exposure to substances by participant in the past year	Y/N	Radio buttons	Reported	x			
25. Occupational exposure to substances participant – type	Type of occupational substances to which the participant was exposed to in the past year	Text	Notes box	Reported	x			
26. Professional activity partner	The current partner being professional active in the past year	Y/N	Radio buttons	Reported	x			
**27. Gross Annual Family Income**	Gross Annual Family Income	<15 000 euro; 15 000 euro - <30 000 euro; 30 000 euro - <45 000 euro; 45 000 euro - <65 000 euro; >=65 000 euro; I don't know / prefer not to say	Radio buttons	Reported	x			
**28. Channel through which the participant was first informed**	Channel through which the participant first heard about the BELpREG study	A healthcare professional informed me (specify type); A friend / family member / neighbour / colleague informed me; Informed by a flyer / poster (specify where); Informed by social media (specify type); Informed by media	Checkboxes, Multiple choice	Reported	x			

		(specify); Informed by an article (specify); Informed by the website apotheek.be; Informed by the website gezondzwangerworden.be; Other (specify)						

Abbreviations : EM = Pregnancy Enrolment Questionnaire; FU = Pregnancy Follow-up Questionnaire; PP 1 = First Postpartum Questionnaire; PP 2 = Second Postpartum Questionnaire

Notes: [1] In case the last menstrual period (LMP) is not directly registered by participants, it is derived from estimated date of delivery (EDD); [2]Structured text field with autocomplete function, linked to NATO database with country names and alpha-2 codes; [3]Structured text field with autocomplete function, linked to the open-source databases of the national post service in Belgium with town names and postal codes

**Category 2: Current pregnancy and health status**								
**Variable**	**Definition**	**Values**	**Field type**	**Source**	**Survey Instruments**			
					**EM**	**FU**	**PP 1**	**PP 2**
**29. Pregnancy status**	Status of still being pregnant	Y/N	Radio buttons			x		
**Gestational age**								
30. Self-reported gestational age	Self-reported current gestational age	Weeks (integer), days (integer – optional) and 'I don't know (yet)' checkbox	Embedded Text boxes and Checkbox	Reported	x	x		
31. Calculated gestational age	Gestational age calculated based on EDD[1]	Number	Calculated field	Derived				
32. Gestational age at identification pregnancy	Gestational age at determination pregnancy	Number	Text box	Reported	x			
**Estimated date of delivery**								
33. Estimated date of delivery (EDD)	Estimated date of delivery	Date (D-M-Y) and 'I don't know yet' checkbox	Text box and Checkbox	Reported	x			
34. Source of the estimated date of delivery	Method used to define the estimated date of delivery	Ultrasound; Calculated based on the first day of the last period; Calculated based on the date of conception (in case of fertility treatment); Other (Specify)	Radio buttons with embedded Text box	Reported	x			
35. Last Menstrual Period (LMP)	Date of the first day of the last menstrual period prior to conception	Date (D-M-Y)	Text box	Reported	x			
**36. Onset of pregnancy**	Onset of the current pregnancy	Spontaneously; After fertility treatment with hormonal stimulation; After fertility treatment without hormonal stimulation	Radio buttons	Reported	x			
**Plurality**								

37. Singleton or multiple pregnancy	Singleton or multiple pregnancy	Singleton; Multiples; I don't know yet	Radio buttons	Reported	x			
38. Number of fetuses	Number of fetuses in current pregnancy	Twins; Triplets, Other (specify - integer)	Radio buttons	Reported	x			
**Pregnancy preparation and planning**								
39. Planned pregnancy	Planned pregnancy	Y/N	Radio buttons	Reported	x			
40. Time until conception	Number of months until conception	Number (+ optional text field to clarify)	Text box	Reported	x			
41. Preparation for pregnancy	Preparation for pregnancy	Y/N	Radio buttons	Reported	x			
42. Type of preparation for pregnancy	How the participant prepared for the current pregnancy	To avoid infections; To have antibodies checked in blood; To check vaccination status; To pursue ideal weight; To get (physical) exercise; To eat healthier; To discuss the use of medicines with an health care professional; To search for online information; To start folic acid intake	Checkboxes, Multiple choice	Reported	x			
43. Expected place of delivery	Expected place of delivery	Hospital; At home; Other (specify); I don't know yet	Radio buttons	Reported	x			
44. Expected hospital of delivery	Expected hospital of delivery	Text - selection from drop-down list[2] or free text	Drop-down list linked to database, Autocomplete	Reported	x			
45. Willingness to breastfeed	Willingness to breastfeed	Y; N; I don't know	Radio buttons	Reported	x			
46. Profession of involved HCPs	Types of HCPs involved in the current pregnancy follow-up	Gynaecologist; Midwife; General Practitioner; Medical specialist (specify type); Psychologist; Other (specify)	Checkboxes, Multiple choice	Reported	x			
47. Name(s) of involved HCPs	Name(s) of HCPs involved in the current pregnancy follow-up	Text - selection from drop-down list[3] or free text	Drop-down list linked to database, Autocomplete	Reported	x			
**Health status participant**								
48. Length	Length at conception, in centimeter	Number	Text box	Reported	x			
49. Weight	Weight at conception, in kilogram	Number	Text box	Reported	x			
50. Body-Mass index (BMI)	BMI, calculated based on length and weight	Number	Calculated field	Derived				

51. Chronic conditions	Chronic conditions (i.e., conditions existing before the start of the pregnancy)	Text - selection from drop-down list[4] or free text - multiple answers possible	Drop-down list linked to database, Autocomplete	Reported	x			
**52. Chronic conditions of the biological father**	Chronic conditions of the biological father, (i.e., existing before the start of the pregnancy)	Text - selection from drop-down list[4] or free text - multiple answers possible - including 'I don't know' option	Drop-down list linked to database, Autocomplete	Reported	x			
**53. Consanguinity**	Mother and biological father have one or more ancestors in common	Y/N	Radio buttons	Reported	x			
**54. Pregnancy complications**	Pregnancy complications (since previous questionnaire)	No complication(s) or disease(s); Severe pregnancy vomiting (hyperemesis gravidarum); Diabetes diagnosed after 20 weeks ('gestational diabetes'); Increased blood pressure after 20 weeks ('Gestational Hypertension'); Pre-eclampsia diagnosed before/during week 34; Pre-eclampsia diagnosed after week 34; HELLP syndrome; Seizures; Growth retardation in the unborn child; Blood clot (thrombosis); Placenta praevia; Placenta abruptio; (severe) bleeding (specify); Depression during pregnancy; Anxiety during pregnancy; Psychosis; Exacerbation of my chronic condition (specify which condition this concerns); Pregnancy cholestasis; Too much amniotic fluid; Too little amniotic fluid; Threatening premature birth; Premature ruptured of membranes (PROM); Premature labour (< 37 weeks); Infection (specify); Other (specify)	Checkboxes with embedded text fields, Multiple choice	Reported	x	x	x	
**Maternal hospital admission**								
55. Hospital admission during pregnancy	Hospital admission since start pregnancy / since previous questionnaire	Y/N	Radio buttons	Reported	x	x	x	
56. Reason for hospital admission during pregnancy	Reason for hospital admission since start pregnancy / since previous questionnaire	Severe pregnancy vomiting (hyperemesis gravidarum); Threatening premature birth; Premature labour (< 37 weeks); Premature rupture of the membranes	Checkboxes with embedded text fields,	Reported	x	x	x	

		(PROM); Pre-eclampsia diagnosed before/during week 34; Pre-eclampsia diagnosed after week 34; HELLP syndrome; Seizures; Growth retardation in the unborn child; Blood clot (thrombosis); Placenta praevia; Placental abruptio; (severe) bleeding; Psychosis; Exacerbation of my chronic condition (specify); Infection (specify); Pregnancy cholestasis; Too much amniotic fluid; Too little amniotic fluid; Cardiac arrhythmias; Other (specify)	Multiple choice					
**Prenatal screening**								
57. NIP test	Performance of a non-invasive prenatal test (NIP test)	Y; N; I don't know	Radio buttons	Reported			x	
58. Congenital anomaly identified during pregnancy	Identification of a congenital anomaly during pregnancy	Y/N	Radio buttons	Reported			x	
59. Source of identification of the congenital anomaly	Source of the identification of the congenital anomaly	NIP test; Ultrasound; Amniocentesis or chorionic villus sampling; Blood test (to determine infections) (specify infection), Other (specify)	Checkboxes, Multiple choice	Reported			x	
**60. Depressive symptoms**	Edinburgh Depression Scale (EDS-10)	10 questions with each time 4 response options	Radio buttons (10)	Reported	x			x

Abbreviations : EM = Pregnancy Enrolment Questionnaire; FU = Pregnancy Follow-up Questionnaire; PP 1 = First Postpartum Questionnaire; PP 2 = Second Postpartum Questionnaire; AC = Auto Complete; MC = Multiple Choice; EDD = Estimated Date of Delivery; LMP = Last Menstrual Period; HCPs = Healthcare professionals; BMI = Body-Mass Index; ICD-11 = International Classification of Diseases 11th Revision; MedDRA = Medical Dictionary for Regulatory Activities; HELLP = Haemolysis, Elevated Liver enzymes and Low Platelets.

Notes: [1] If the estimated date of delivery (EDD) is not known, the calculation of gestational age is based on the last menstrual period (LMP); [2] Structured text field with autocomplete function, linked to the open-source database with names and cities of hospitals in Belgium; [3]Structured text field with autocomplete function, linked to the open-source RIZIV/FGOV database with registered HCPs in Belgium, including their name, profession, qualification and work address; [4]Structured text field with autocomplete function, linked to a self-developed list of the most frequently reported disorders linked to their respective ICD-11 and MedDRA classification.

**Category 3: Maternal-obstetric history**								
**Variable**	**Definition**	**Values**	**Field type**	**Source**	**Survey Instruments**			
					**EM**	**FU**	**PP 1**	**PP 2**
**61. Previous pregnancy**	Previously having been pregnant	Y/N	Radio buttons	Reported	x			
**62. Number of previous pregnancies**	Number of previous pregnancies	Integer	Text box	Reported	x			
**63. Gravidity**	Gravidity	Primigravida; Multigravida	Calculated field	Derived				
**History of miscarriage**								
64. Previous miscarriage	Previous spontaneous interruption of pregnancy before the end of week 22, calculated from the first day of the last menstrual period	Y/N	Radio buttons	Reported	x			
65. Number of previous miscarriages	Number previous miscarriages	Integer	Text box	Reported	x			
66. Gestational age at previous miscarriage(s)	Gestational age at previous miscarriage(s)	Text	Text box	Reported	x			
**History of elective termination of pregnancy (ETOP)**								
67. Previous ETOP	Previous ETOP	Y/N	Radio buttons	Reported	x			
68. Number of previous ETOPs	Number of previous ETOPs	Integer	Text box	Reported	x			
69. Gestational age at ETOP(s)	Gestational age at previous ETOP(s)	Text	Text box	Reported	x			
70. Reason of ETOP(s)	Reason of ETOP(s)	The pregnancy(ies) was (were) terminated due to medical reasons for myself; The pregnancy(ies) was (were) terminated for medical reasons in the embryo/fetus; The pregnancy(ies) was (were) terminated at my personal request	Radio buttons	Reported	x			
71. Medical reason of ETOP(s)	The actual medical reason of ETOP(s)	Text	Notes box	Reported	x			
**History of stillbirth**								
72. Previous stillbirth	Death of an unborn infant in the womb after 22 weeks of gestation	Y/N	Radio buttons	Reported	x			
73. Gestational age at stillbirth(s)	Gestational age at stillbirth(s)	Text	Text box	Reported	x			

74. Congenital anomaly	Identification of a congenital anomaly in the stillborn fetus	Y (specify) / N	Radio buttons with embedded Text box	Reported	x			
**75. Parity**	Parity	Nullipara, Primipara, Multipara	Calculated field	Derived				
**76. History of fertility treatment**	Fertility treatment before the current pregnancy	Y/N	Radio buttons	Reported	x			
**History of pregnancy / delivery complication(s)**								
77. History of pregnancy complications	Pregnancy complications during previous pregnancy(ies)	No complication(s); Severe pregnancy vomiting (hyperemesis gravidarum); Diabetes diagnosed after 20 weeks ('gestational diabetes'); Increased blood pressure after 20 weeks ('gestational hypertension'); Pre-eclampsia diagnosed before/during week 34; Pre-eclampsia diagnosed after week 34; HELLP syndrome; Seizures; Growth retardation in the unborn child; Blood clot (thrombosis); Placenta praevia; Placental abruptio; (severe) bleeding (specify); Depression during pregnancy; Anxiety during pregnancy; Psychosis; Exacerbation of my chronic condition (specify which condition this concerns); Pregnancy cholestasis; Too much amniotic fluid; Too little amniotic fluid; Threatening premature birth; Premature rupture of the membranes (PROM); Premature labour (< 37 weeks); Infection (specify); Other (specify)	Checkboxes with embedded text fields, Multiple choice	Reported	x			
78. History of delivery complications	Delivery complications during previous pregnancy(ies)	No Complications; Giving birth before 32 weeks; Giving birth between 32 and 37 weeks; New-born with a low birth weight (< 2500 grams); New-born with a high birth weight (>=4000 grams); Blood clot (thrombosis); Coloured amniotic fluid; Shoulder dystocia; No birth of the placenta within 30 minutes after birth of	Checkboxes with embedded text fields, Multiple choice	Reported	x			

		the new-born ('placenta retention'); Ruptured uterus ('uterine rupture'); Oxygen deficiency in the child during childbirth; Amniotic fluid embolism; Sepsis; Acute renal failure; Severe blood loss after delivery (>1000mL); High blood pressure; Cardiac arrhythmias; Postpartum Depression; Anxiety after childbirth; Post-traumatic stress syndrome after childbirth; Psychosis; Other (specify)						
**Predisposition to congenital anomalies**								
79. Congenital anomaly during a previous pregnancy	Identification of congenital anomaly(ies) in a previous pregnancy of the participant and/or biological father of the unborn child	Y; N; I don't know	Radio buttons	Reported	x			
80. Description of congenital anomaly(ies) in a previous pregnancy	Description of the congenital anomaly(ies) in a previous pregnancy of the participant and/or biological father of the unborn child	Text	Notes box	Reported	x			
81. Cause of congenital anomaly(ies) in a previous pregnancy	Cause of the congenital anomaly(ies) in a previous pregnancy of the participant and/or biological father of the unborn child	There is no known genetic cause; There is a known genetic cause; A suspected genetic cause is known; I don't know; Other (specify)	Checkboxes, Multiple choice	Reported	x			
82. Congenital anomaly in the participant	Identification of congenital anomaly(ies) in the participant	Y/N	Radio buttons	Reported	x			
83. Description of congenital anomaly(ies) in the participant	Description of the congenital anomaly(ies) in the participant	Text	Notes box	Reported	x			
84. Cause of congenital anomaly(ies) in the participant	Cause of the congenital anomaly(ies) in the participant	There is no known genetic cause; There is a known genetic cause; A suspected genetic cause is known; Other (specify)	Checkboxes, Multiple choice	Reported	x			
85. Congenital anomaly in the father	Identification of congenital anomaly(ies) in the biological father	Y; N; I don't know	Radio buttons	Reported	x			
86. Description of congenital anomaly(ies) in the father	Description of the congenital anomaly(ies) in the biological father	Text	Notes box	Reported	x			
87. Cause of congenital anomaly(ies) in the father	Cause of the congenital anomaly(ies) in the biological father	There is no known genetic cause; There is a known genetic cause; A suspected	Checkboxes, Multiple choice	Reported	x			

		genetic cause is known; I don't know; Other (specify)						
88. Congenital anomaly(ies) in a parent or sibling	Identification of congenital anomaly(ies) in parent or sibling of the participant and/or biological father	Y; N; I don't know	Radio buttons	Reported	x			
89. Description of congenital anomaly(ies) in a parent or sibling	Description of congenital anomaly(ies) in a parent or sibling of mother or biological father, including in whom this was diagnosed	Text	Notes box	Reported	x			
90. Cause of congenital anomaly(ies) in a parent or sibling	Cause of congenital anomaly(ies) in a parent or sibling of mother or biological father	There is no known genetic cause; There is a known genetic cause; A suspected genetic cause is known; I don't know; Other (specify)	Checkboxes, Multiple choice	Reported	x			
**Predisposition to developmental disorders**								
91. Developmental disorder during previous pregnancy	Identification of developmental disorder(s) in a previous pregnancy of the participant and/or the biological father of the unborn child	Y; N; I don't know	Radio buttons	Reported	x			
92. Description developmental disorder(s) during previous pregnancy	Description of developmental disorder(s) in a previous pregnancy of the participant and/or the biological father of the unborn child	Text	Notes box	Reported	x			
93. Developmental disorder in the participant	Identification of developmental disorder(s) in the participant	Y/N	Radio buttons	Reported	x			
94. Description of developmental disorder(s) in the participant	Description of developmental disorder(s) in the participant	Text	Notes box	Reported	x			
95. Developmental disorder in the father	Identification of developmental disorder(s) in the biological father	Y; N; I don't know	Radio buttons	Reported	x			
96. Description of developmental disorder(s) in the father	Descriptions of developmental disorder(s) in the biological father	Text	Notes box	Reported	x			
97. Developmental disorder in a parent or sibling	Identification of developmental disorder(s) in a parent or sibling of mother or biological father	Y; N; I don't know	Radio buttons	Reported	x			
98. Description of developmental disorder(s) in a parent or sibling	Descriptions of developmental disorder(s) in a parent or sibling of mother or biological father, including	Text	Notes box	Reported	x			

	the notification in whom this was diagnosed							
**Predisposition to motor disorders**								
99. Motor disorder during a previous pregnancy	Identification of motor disorder(s) in a previous pregnancy of the participant and/or biological father of the unborn child	Y; N; I don't know	Radio buttons	Reported	x			
100. Description motor disorder(s) during a previous pregnancy	Description of motor disorder(s) in a previous pregnancy of the participant and/or the biological father of the unborn child	Text	Notes box	Reported	x			
101. Motor disorder in the participant	Identification of motor disorder(s) in the participant	Y/N	Radio buttons	Reported	x			
102. Description motor disorder(s) in the participant	Description of motor disorder(s) in the participant	Text	Notes box	Reported	x			
103. Motor disorder in the father	Identification of motor disorder(s) in the biological father	Y; N; I don't know	Radio buttons	Reported	x			
104. Description motor disorder(s) in the father	Description of motor disorder(s) in the biological father	Text	Notes box	Reported	x			

Abbreviations : EM = Pregnancy Enrolment Questionnaire; FU = Pregnancy Follow-up Questionnaire; PP 1 = First Postpartum Questionnaire; PP 2 = Second Postpartum Questionnaire; ETOP = Elective Termination Of Pregnancy; HELLP = Haemolysis, Elevated Liver enzymes and Low Platelets.

Category 4: Use of medicines, folic acid / pregnancy vitamins and other health products								
**Variable**	**Definition**	**Values**	**Field type**	**Source**	**Survey Instruments**			
					**EM**	**FU**	**PP 1**	**PP 2**
**Medication use during pregnancy** - The following questions are repeated for each reported medicine								
**105. Name of the medicine**	Name of the medicine used since the start of the pregnancy (i.e., in the enrolment questionnaire) or since completing the previous questionnaire (i.e., in a follow-up questionnaire) or since completing the previous questionnaire and the end of the pregnancy (i.e., in the postpartum 1 questionnaire)	Text - selection from drop-down list[1] or free text	Drop-down list linked to database, Autocomplete	Reported	x	x	x	
**106. Modality of medicine use**	How was/has the medicine been used	a) Daily and still (e.g., thyroid hormone);<br>b) Occasionally or as needed (e.g., a painkiller taken occasionally, or an injection that is regularly given);<br>c) During a certain period and not anymore (e.g., an antibiotic that was used for 5 days)	Radio buttons	Reported	x	x	x	
Medication use during pregnancy – modality 'a) Daily and still'								
107. How many times a day	How many times the medicine has been used a day	1x per day; 2x a day; 3x per day; Other (specify)	Radio buttons	Reported	x	x	x	
108. Quantity per intake – number	Number of units per intake	½; 1; 2; 3; 4; Other (specify)	Text box	Reported	x	x	x	
109. Quantity per intake – unit	Unit of intake	Capsule(s) or tablet(s); Teaspoon(s) (5mL); Tablespoon(s) (15mL); Spray(s) (e.g., per nostril); Puff(s); Drop(s) (e.g., per nostril or eye); Syringe(s) / ampoule(s); Units; Suppository(s) / ovule(s); Fingertip(s) (with ointment/cream/gel); Patch(s); Sachet(s); Other (specify)	Dropdown	Reported	x	x	x	
110. Start of use	Timing of medication use initiation	Before this pregnancy; During this pregnancy	Radio buttons	Reported	x			
111. Start of use during pregnancy – date	Start date of medication use during this pregnancy	Date (DD-MM-YYYY)	Text box	Reported	x	x	x	

112. Continuation of use	Continuation of a previously registered medicine under the modality ‘Daily and still’[3].	Yes; I no longer use this medicine (if so, when did you stop); The use of this medicine has changed (if so, date of change and variables 107-109 are questioned)	Radio buttons	Reported		x	x	
Medication use during pregnancy - modality 'b) Occasionally or as needed'								
113. Number of days when medicine was used	Number of days when the medicine was used since the start of the pregnancy (i.e., in the enrolment questionnaire) or since completing the previous questionnaire (i.e., in a follow-up questionnaire) or since completing the previous questionnaire and the end of the pregnancy (i.e., in the postpartum 1 questionnaire)	- During the first trimester (from week 1 to the end of week 12): integer<br>- During the second trimester (from week 13 to the end of week 26): integer<br>- During the third trimester (from week 27 until the end of the pregnancy): integer	Text box (3)	Reported	x	x	x	
114. How many times a day	How many times the medicine was used a day	1x per day; 2x a day; 3x per day; Variable (specify); Other (specify)	Radio buttons	Reported	x	x	x	
115. Quantity per intake - number	Number of units per intake	1/2; 1; 2; 3; 4; Other (specify)	Text box	Reported	x	x	x	
116. Quantity per intake - unit	Unit of intake	Capsule(s) or tablet(s); Teaspoon(s) (5mL); Tablespoon(s) (15mL); Spray(s) (e.g., per nostril); Puff(s); Drop(s) (e.g., per nostril or eye); Syringe(s) / ampoule(s); Units; Suppository(s) / ovule(s); Fingertip(s) (with ointment/cream/gel); Patch(s); Sachet(s); Other (specify)	Dropdown	Reported	x	x	x	
117. Start of use during pregnancy - date	Start date of medication use during this pregnancy	Date (DD-MM-YYYY)	Text box	Reported	x	x	x	
118. Use in 6 months before start pregnancy	Use of the medicine in the 6 months before the start of the pregnancy	Y/N	Radio buttons	Reported	x			
Medication use during pregnancy - modality 'c) During a certain period and not anymore'								
119. How many times a day	How many times the medicine was used a day	1x per day; 2x a day; 3x per day; Variable (specify); Other (specify)	Radio buttons	Reported	x	x	x	
120. Quantity per intake - number	Number of units per intake	1/2; 1; 2; 3; 4; Other (specify)	Text box	Reported	x	x	x	
121. Quantity per intake - unit	Unit of intake	Capsule(s) or tablet(s); Teaspoon(s) (5mL); Tablespoon(s) (15mL); Spray(s)	Dropdown	Reported	x	x	x	

		(e.g., per nostril); Puff(s); Drop(s) (e.g., per nostril or eye); Syringe(s) / ampoule(s); Units; Suppository(s) / ovule(s); Fingertip(s) (with ointment/cream/gel); Patch(s); Sachet(s); Other (specify)						
122. Start of use	Timing of medication use initiation	Before this pregnancy; During this pregnancy	Radio buttons	Reported	x	x	x	
123. Start of use during pregnancy - date	Start date of medication use during this pregnancy	Date (DD-MM-YYYY)	Text box	Reported	x	x	x	
124. Stop of use	Stop date of medication use	Date (DD-MM-YYYY)	Text box	Reported	x	x	x	
**125. Indication of medication use**	Indication of the medication use, as reported by the participant	Text - selection from drop-down list[3] or free text	Drop-down list linked to database, Autocomplete	Reported	x	x	x	
**4.2 Use of folic acid and pregnancy vitamins during pregnancy** - The following questions are repeated for each reported folic acid product and pregnancy vitamin								
**126. Name of the folic acid product or pregnancy vitamin**	Name of the folic acid product or pregnancy vitamin used currently (i.e., in the enrolment questionnaire) or since completing the previous questionnaire (i.e., in a follow-up questionnaire) or since completing the previous questionnaire and the end of the pregnancy (i.e., in the postpartum 1 questionnaire)	Text – selection from drop-down list[1] or free text	Drop-down list linked to database, Autocomplete	Reported	x	x	x	
127. Start of use	Start date of using the folic acid product or pregnancy vitamin	Date (DD-MM-YYYY)	Text box	Reported	x	x	x	
128. How many times a day	How many times the folic acid product or pregnancy vitamin was/has been used	1x per day; Other (specify)	Radio buttons	Reported	x	x	x	
129. Stop of use	Stop date of using the folic acid product or pregnancy vitamin	Date (DD-MM-YYYY)	Text box	Reported		x	x	
130. Continuation of use	Continuation of a previously registered folic acid product or pregnancy vitamin[4]	Yes; I no longer use this product (if so, when did you stop); The use of this product has changed (if so, date of change and variable 128 is questioned)	Radio buttons	Reported		x	x	
**131. Name of a folic acid product or pregnancy vitamin used earlier in pregnancy**	Name of a folic acid product or pregnancy vitamin used earlier in this pregnancy	Text - selection from drop-down list[1] or free text	Drop-down list linked to database, Autocomplete	Reported	x			

132. How many times a day	How many times the folic acid product or pregnancy vitamin was used	1x per day; Other (specify)	Radio buttons	Reported	x			
133. Start of use	Start date of using the folic acid product or pregnancy vitamin	Date (DD-MM-YYYY)	Text box	Reported	x			
134. Stop of use	Stop date of using the folic acid product or pregnancy vitamin	Date (DD-MM-YYYY)	Text box	Reported	x			
**4.3 Health products' use during pregnancy** - The following questions are repeated for each reported health product								
**135. Name of the health product**	Name of the health product used since the start of the pregnancy (i.e., in the enrolment questionnaire) or since completing the previous questionnaire (i.e., in a follow-up questionnaire) or since completing the previous questionnaire and the end of the pregnancy (i.e., in postpartum 1 questionnaire)	Text - selection from drop-down list[1] or free text	Drop-down list linked to database, Autocomplete	Reported	x	x	x	
**136. Modality of health product use**	How was/has the health product been used	a) Daily and still (e.g., thyroid hormone);<br>b) Occasionally or as needed (e.g., a painkiller taken occasionally, or an injection that is given regularly);<br>c) During a certain period and not anymore (e.g., an antibiotic that was used for 5 days)	Radio buttons	Reported	x	x	x	
Health products' use during pregnancy - modality 'a) Daily and still'								
137. How many times a day	How many times the health product has been used a day	1x per day; 2x a day; 3x per day; Other (specify)	Radio buttons	Reported	x	x	x	
138. Quantity per intake - number	Number of units per intake	1/2; 1; 2; 3; 4; Other (specify)	Text box	Reported	x	x	x	
139. Quantity per intake - unit	Unit of intake	Capsule(s) or tablet(s); Teaspoon(s) (5mL); Tablespoon(s) (15mL); Spray(s) (e.g., per nostril); Puff(s); Drop(s) (e.g., per nostril or eye); Syringe(s) / ampoule(s); Units; Suppository(s) / ovule(s); Fingertip(s) (with ointment/cream/gel); Patch(s); Sachet(s); Other (specify)	Dropdown	Reported	x	x	x	
140. Start of use	Timing of the initiation of the use of the health product	Before this pregnancy; During this pregnancy	Radio buttons	Reported	x			

141. Start of use during pregnancy - date	Start date of using the health product during this pregnancy	Date (DD-MM-YYYY)	Text box	Reported	x	x	x	
142. Continuation of use	Continuation of a previously registered health product under the modality 'Daily and still'[2].	Yes; I no longer use this product (if so, when did you stop); The use of this product has changed (if so, date of change and variables 137-139 is questioned)	Radio buttons	Reported		x	x	
Health products' use during pregnancy - modality 'b) Occasionally or as needed'								
143. Number of days when heath product used	Number of days when the health product was used since the start of the pregnancy (i.e., in the enrolment questionnaire) or since completing the previous questionnaire (i.e., in a follow-up questionnaire) or since completing the previous questionnaire and the end of the pregnancy (i.e., in the postpartum 1 questionnaire)	- During the first trimester (from week 1 to the end of week 12): integer<br>- During the second trimester (from week 13 to the end of week 26): integer<br>- During the third trimester (from week 27 until the end of the pregnancy): integer	Text box (3)	Reported	x	x	x	
144. How many times a day	How many times the health product was used a day	1x per day; 2x a day; 3x per day; Variable (specify); Other (specify)	Radio buttons	Reported	x	x	x	
145. Quantity per intake - number	Number of units per intake	1/2; 1; 2; 3; 4; Other (specify)	Text box	Reported	x	x	x	
146. Quantity per intake - unit	Unit of intake	Capsule(s) or tablet(s); Teaspoon(s) (5mL); Tablespoon(s) (15mL); Spray(s) (e.g., per nostril); Puff(s); Drop(s) (e.g., per nostril or eye); Syringe(s) / ampoule(s); Units; Suppository(s) / ovule(s); Fingertip(s) (with ointment/cream/gel); Patch(s); Sachet(s); Other (specify)	Dropdown	Reported	x	x	x	
147. Start of use during pregnancy - date	Start date of the use of the health product during this pregnancy	Date (DD-MM-YYYY)	Text box	Reported	x	x	x	
148. Use in 6 months before start pregnancy	Use of the health product in the 6 months before the start of the pregnancy	Y/N	Radio buttons	Reported	x			
Health products' use during pregnancy - modality 'c) During a certain period and not anymore'								
149. How many times a day	How many times the health product was used a day	1x per day; 2x a day; 3x per day; Variable (specify); Other (specify)	Radio buttons	Reported	x	x	x	
150. Quantity per intake - number	Number of units per intake	1/2; 1; 2; 3; 4; Other (specify)	Text box	Reported	x	x	x	

151. Quantity per intake - unit	Unit of intake	Capsule(s) or tablet(s); Teaspoon(s) (5mL); Tablespoon(s) (15mL); Spray(s) (e.g., per nostril); Puff(s); Drop(s) (e.g., per nostril or eye); Syringe(s) / ampoule(s); Units; Suppository(s) / ovule(s); Fingertip(s) (with ointment/cream/gel); Patch(s); Sachet(s); Other (specify)	Dropdown	Reported	x	x	x	
152. Start of use	Timing of the initiation of the use of the health product	Before this pregnancy; During this pregnancy	Radio buttons	Reported	x	x	x	
153. Start of use during pregnancy - date	Start date of the use of the health product during this pregnancy	Date (DD-MM-YYYY)	Text box	Reported	x	x	x	
154. Stop of use	Stop date of the use of the health product	Date (DD-MM-YYYY)	Text box	Reported	x	x	x	
**155. Indication of the health product**	Indication of the use of the health product, as reported by the participant	Text - selection from drop-down list[3] or free text	Drop-down list linked to database, Autocomplete	Reported	x	x	x	
**Other medicines and health products used in the 6 months before the start of the pregnancy**								
**156. Name of the medicine or health product**	Name of the medicine or health product used in the 6 months before the start of the pregnancy	Text - selection from drop-down list[1] or free text	Drop-down list linked to database, Autocomplete	Reported	x			
157. Stop of use	Stop date of the use of the medicine or health product (estimation)	Date (DD-MM-YYYY)	Text box	Reported	x			
**Paternal medication use**								
**158. Name of the medicine or health product**	Name of the medicine or health product used by the biological father in the 3 months before the start of the pregnancy	Text - selection from drop-down list[1] or free text - including 'I don't know' option	Drop-down list linked to database, Autocomplete	Reported	x			
**Medication use postpartum**								
**159. Name of the medicine or health product**	Name of the medicine or health product used since childbirth	Text - selection from drop-down list[1] or free text	Drop-down list linked to database, Autocomplete	Reported			x	x

Abbreviations : EM = Pregnancy Enrolment Questionnaire; FU = Pregnancy Follow-up Questionnaire; PP 1 = First Postpartum Questionnaire; PP 2 = Second Postpartum Questionnaire; ATC = Anatomical Therapeutic Chemical; CNK = National Code Number; VMP = Virtual Medicinal Product; AMP = Actual Medicinal Product; NMP = Non Medicinal Product; ICD-11 = International Classification of Diseases 11th Revision; MedDRA = Medical Dictionary for Regulatory Activities.

Notes: [1]Structured text field with autocomplete function, including pictures of the products, linked to a database of available medicines and medicinal products in Belgium. Additional information of the product is derived from the linked database, including ATC code, CNK, VMP, VMP group, Substance, Substance strength, Route of drug administration, Pharmaceutical form, Medication / non-medication status; [2]If a medicine or health product was registered as 'Daily and still' in the previous questionnaire, this medicine will be shown with the reported details of use in the next questionnaire. This question is always shown first, after which other products can be registered; [3]Structured text field with autocomplete function, linked to a self-developed list of the most frequent reported indications / conditions that are linked to their respective ICD-11 and MedDRA classification; [4]If a folic acid product or pregnancy vitamin was registered in the previous questionnaire, this medicine will be shown with the reported details of use in the next questionnaire. This question is always shown first, after which other products can be registered.

Category 5: Substance use

Variable	Definition	Values	Field type	Source	Survey Instruments			
					EM	FU	PP 1	PP 2
**Smoking**								
160. Smoking - ever	Smoking, ever done (normal cigarette or e-cigarette)	Y/N	Radio buttons	Reported	x			
161. Smoking - past year	Smoking in the past year	Y/N	Radio buttons	Reported	x			
162. Smoking - in pregnancy	Smoking during this pregnancy	Y/N	Radio buttons	Reported	x			
163. Timing of smoking cessation	Timing of smoking cessation	In the past year; Since trying to get pregnant; Since I knew I was pregnant	Radio buttons	Reported	x			
164. Smoking status throughout pregnancy	Smoking status throughout pregnancy	Y/N	Radio buttons	Reported	x	x		
165. Timing of smoking cessation in pregnancy	Gestational age upon smoking cessation	Number	Text box	Reported	x	x		
166. Amount of smoking in pregnancy	Daily amount of smoking in this pregnancy	1-10 cigarettes/day; 11-20 cigarettes/day; 21-30 cigarettes/day; >30 cigarettes/day	Radio buttons	Reported	x	x		
167. Postpartum smoking status	Smoking since delivery	Y/N	Radio buttons	Reported			x	x
168. Frequency of smoking since delivery	Frequency of smoking since delivery	1-10 cigarettes/day; 11-20 cigarettes/day; 21-30 cigarettes/day; >30 cigarettes/day	Radio buttons	Reported			x	x
169. Exposure to second-hand smoking during pregnancy	Smoking by someone who lives together with the pregnant participant	Y/N	Radio buttons	Reported	x			
170. Amount of second-hand smoking during pregnancy	Daily amount of smoking by someone who lives together with the pregnant participant	1-10 cigarettes/day; 11-20 cigarettes/day; 21-30 cigarettes/day; >30 cigarettes/day	Radio buttons	Reported	x			

171. Second-hand smoking in-house during pregnancy	Smoking in-house by someone who lives together with the pregnant participant	Y/N	Radio buttons	Reported	x			
**Alcohol use**								
172. Alcohol use - past year	Use of alcohol in the past year	Y/N	Radio buttons	Reported	x			
173. Alcohol use - in pregnancy	Use of alcohol during this pregnancy	Y/N	Radio buttons	Reported	x			
174. Timing of alcohol cessation	Timing of alcohol cessation	In the past year; Since trying to get pregnant; Since I knew I was pregnant	Radio buttons	Reported	x			
175. Alcohol use status throughout pregnancy	Alcohol use status throughout pregnancy	Y/N	Radio buttons	Reported	x	x		
176. Timing of alcohol use cessation in pregnancy	Gestational age upon alcohol use cessation	Number	Text box	Reported	x	x		
177. Alcohol use after delivery	Alcohol use after delivery	Y/N	Radio buttons	Reported			x	x
178. Frequency of drinking alcohol in pregnancy	Frequency of drinking alcohol in this pregnancy	Monthly or less; 2-4 times a month; 2-3 times a week; 4 or more times a week	Radio buttons	Reported	x	x		
179. Frequency of drinking alcohol after delivery	Frequency of drinking alcohol after delivery	Monthly or less; 2-4 times a month; 2-3 times a week; 4 or more times a week	Radio buttons	Reported			x	x
180. Amount of alcohol per occasion	Number of standard glasses of alcohol used per occasion. 1 standard glass is the amount of a drink that is usually served in a pub or at a restaurant, e.g., 25cl beer or 10cl wine	<1; 1 or 2; 3 or 4; 5 or 6; 7 or 9; 10 or more	Radio buttons	Reported	x	x	x	x
181. Occurrence of binge drinking	Occurrence of using 6 or more standard glasses of alcohol per occasion	Never; Less than monthly; Monthly; Weekly; Daily or almost daily	Radio buttons	Reported	x	x	x	x
182. Alcohol use in the close environment during pregnancy	Daily use of alcohol by someone who lives together with the pregnant participant	Y/N	Radio buttons	Reported	x			
**Cannabis and other illicit drugs**								
183. Cannabis and other illicit drug use - ever	Cannabis and other illicit drug use, ever used	Y/N	Radio buttons	Reported	x			
184. Cannabis and other illicit drug use - past year	Use of cannabis and other illicit drugs in the past year	Y/N	Radio buttons	Reported	x			
185. Cannabis and other illicit drug use - in pregnancy	Use of cannabis and other illicit drugs in this pregnancy	Y/N	Radio buttons	Reported	x			

186. Timing of cessation of use of cannabis and other illicit drugs	Timing of cessation of the use of cannabis and other illicit drugs	In the past year; Since trying to get pregnant; Since I knew I was pregnant	Radio buttons	Reported	x			
187. Type of drugs used	Type of drugs used in pregnancy or after delivery, i.e., cannabis (hasj, weed, marihuana), XTC (MDMA), amphetamines (speed,…), hallucinogens (LSD, magic mushrooms), cocaine, ketamine, heroine, GHB, other (specify)	Never used; Less than once a month; Multiple times a month; Once a week; Multiple times a week; Daily	Matrix with radio buttons	Reported	x	x	x	x
188. Drug use in the close environment during pregnancy	Drug use by someone who lives together with the pregnant participant	Y/N	Radio buttons	Reported	x			

Abbreviations : EM = Pregnancy Enrolment Questionnaire ; FU = Pregnancy Follow-up Questionnaire ; PP 1 = First Postpartum Questionnaire ; PP 2 = Second Postpartum Questionnaire.

**Category 6: Pregnancy outcomes**								
**Variable**	**Definition**	**Values**	**Field type**	**Source**	**Survey Instruments**			
					**EM**	**FU**	**PP 1**	**PP 2**
**189. Pregnancy outcome**	Identification of the outcome of the current pregnancy	- I gave birth to a live birth child/children;<br>- I had a miscarriage (i.e., a spontaneous termination of pregnancy before the end of pregnancy week 22);<br>- I had an ectopic pregnancy;<br>- My pregnancy was terminated for medical reasons or at my request;<br>- The fetus has died in the womb, after 22 weeks gestational age (i.e., stillbirth)	Checkboxes, Multiple choice	Reported			x	
In case of 'live birth child(ren)'								
**Plurality[1]**								
190. Singleton or multiple pregnancy	Singleton or multiple pregnancy	Singleton; Multiple	Radio buttons	Reported			x	
191. Number of children	Number of children born after the current pregnancy	Twins; Triplets, Other (specify - integer)	Radio buttons	Reported			x	
**Delivery**								
192. Actual date of delivery	Date of delivery	Date (D-M-Y)	Text box	Reported			x	

193. Gestational age at delivery	Gestational age at delivery	Weeks (integer) and days (integer)	Text box	Reported			x	
194. Expected date of delivery	Expected date of delivery	Date (D-M-Y)	Text box	Reported			x	
195. Mode of delivery	Mode of delivery	Spontaneous vaginal delivery; Assisted (instrumental) vaginal delivery; Planned caesarean section; Unplanned caesarean section; Other (specify)	Radio buttons	Reported			x	
196. Onset of labor	Onset / start of labor	Spontaneous; Initiated (+reason); Not (yet) in labor at the time of the planned caesarean section; Other (specify)	Radio buttons	Reported			x	
197. Reason of caesarean section	Reason of the caesarean section	Previous caesarean section; Non-progressive delivery; Congenital anomaly of the neonate (e.g., heart problem, spina bifida...); Fetal distress (e.g., poor heart tones) (specify problems); Medical condition of the pregnant woman (e.g., preeclampsia) (specify problems); Extreme prematurity; Abnormal position of the baby; Placenta praevia; Other (specify)	Checkboxes with embedded text fields, Multiple choice	Reported			x	
198. Complications after delivery	Complications or medical problems that occurred in the first 72 hours after delivery	No complications; Meconium stained amniotic fluid; Shoulder dystocia; Severe postpartum blood loss (>1000mL); Retained placenta; Uterine rupture; Oxygen deficiency in the neonate during childbirth; Amniotic fluid embolism; Infection (specify type); Sepsis; Acute renal failure; Thrombosis; High blood pressure; Cardiac arrhythmias; Psychosis; Other (specify) - Including possibility to add free text	Checkboxes with embedded text fields, Multiple choice - Notes box	Reported			x	
199. Maternal blood transfusion	Maternal blood transfusion after delivery	Y/N	Radio buttons	Reported			x	
200. Admission to a maternal intensive care unit	Admission of the mother to an intensive care unit after delivery	Y/N	Radio buttons	Reported			x	
201. Reason of admission to a maternal intensive care unit	Reason for the admission of the mother to a maternal intensive care unit after delivery	Text	Notes box	Reported			x	

202. Place of delivery	Place of delivery	Hospital; At home; Other (specify)	Radio buttons	Reported			x	
203. Hospital of delivery	City of the hospital of delivery	Text - selection from drop-down list[2] or free text	Drop-down list linked to database, Autocomplete	Reported			x	
**Postpartum complications**								
204. Postpartum complications	Maternal complications/medical problems that occurred in the first 6 weeks postpartum	No complications; (severe) bleeding (specify); Retained placenta; High blood pressure; Thrombosis/embolism; Mastitis; Breast abscess; Endometritis; Urinary tract infection; Wound problems/infection; Sepsis; Postnatal depression; Psychosis; Flare up of my chronic condition (specify which condition this concerns); Other (specify) - Including possibility to add free text	Checkboxes with embedded text fields, Multiple choice - Notes box	Reported			x	x
205. Maternal hospital admission postpartum	Hospital admission of the mother in the first 6 weeks postpartum	Y/N	Radio buttons	Reported			x	x
206. Reason of maternal hospital admission postpartum	Reason of hospital admission of the mother in the first 6 weeks postpartum	No complications; (severe) bleeding (specify); Retained placenta; High blood pressure; Thrombosis/embolism; Mastitis; Breast abscess; Endometritis; Urinary tract infection; Wound problems/infection; Sepsis; Postnatal depression; Psychosis; Flare up of my chronic condition (specify which condition this concerns); Other (specify) - Including possibility to add free text	Checkboxes with embedded text fields, Multiple choice - Notes box	Reported			x	x
In case of a 'miscarriage'								
**207. Gestational age at miscarriage**	Gestational age at miscarriage	Weeks (text)	Text box	Reported			x	
**208. Plurality**	Singleton or multiple pregnancy	Y/N	Radio buttons	Reported			x	
**209. Reason of the miscarriage**	Indication of a possible reason of the miscarriage	Y/N	Radio buttons	Reported			x	
**210. Description of the reason of the miscarriage**	Description of the possible reason of the miscarriage	Text	Notes box	Reported			x	
In case of an 'elective termination of pregnancy (ETOP)'								

**211. Gestational age at ETOP**	Gestational age at ETOP	Weeks (text)	Text box	Reported			x	
**212. Reason of the ETOP**	Reason of the ETOP	The pregnancy was terminated due to maternal medical reasons; The pregnancy was terminated due to medical reasons in the embryo/fetus; The pregnancy was terminated at my personal request	Radio buttons	Reported			x	
**213. Medical reason ETOP**	Medical reason of the ETOP	Text	Notes box	Reported			x	
In case of a 'stillbirth'								
**214. Gestational age at stillbirth**	Gestational age at stillbirth	Text	Text box	Reported			x	
**215. Identification of a congenital anomaly**	Presence of a congenital anomaly in the stillborn fetus	Y (specify) / N	Radio buttons with embedded Text box	Reported			x	
**216. Name of the involved doctor(s) or midwife(s)** ***(asked to all participants)***	Name of the doctor(s) and/or midwife(s) involved in the pregnancy follow-up	Text - selection from drop-down list[3] or free text	Drop-down list linked to database, Autocomplete	Reported			x	

Abbreviations : EM = Pregnancy Enrolment Questionnaire; FU = Pregnancy Follow-up Questionnaire; PP 1 = First Postpartum Questionnaire; PP 2 = Second Postpartum Questionnaire; ETOP = Elective termination Of Pregnancy; NIP = Non-Invasive Prenatal Screening

Notes: [1]In case of a multiple pregnancy, the neonatal outcome fields are multiplied (see category 7); [2]Structured text field with autocomplete function, linked to the open-source hospital database with names and cities of hospitals in Belgium; [3]Structured text field with autocomplete function, linked to the open-source RIZIV/FGOV database with registered HCPs in Belgium, including their name, profession, qualification and work address.

Category 7: Neonatal outcomes[1]								
**Variable**	**Definition**	**Values**	**Field type**	**Source**	**Survey Instruments**			
					**EM**	**FU**	**PP 1**	**PP 2**
**Biometry at birth**								
217. Birth weight	Birth weight in gram	Number	Text box	Reported			x	
218. Birth length	Birth length in centimeter	Number	Text box	Reported			x	
219. Head circumference	Head circumference of the infant at birth	Number - including 'I don't know option'	Text box and Checkbox	Reported			x	
**220. Sex**	Infant sex	Male; Female; Other (specify)	Text box	Reported			x	
**221. Apgar score**	Apgar score after 1, 5 and 10 minutes	Integer	Text box (3)	Reported			x	
**222. Nationality infant**	Nationality infant	Belgian; Dutch; French; German; Moroccan; Romanian; Turkish; Polish; Syrian; Other (specify[2])	Checkboxes, Multiple choice	Reported			x	
**Congenital anomalies**								
223. Congenital anomaly	Identification of a congenital anomaly	Y/N	Radio buttons	Reported			x	x
224. Congenital anomaly - Description	Description of the congenital anomaly	Text	Text box	Reported			x	x
225. Congenital anomaly - cause	Possible cause of the congenital anomaly	There is no known genetic cause; There is a known genetic cause; A suspected genetic cause is known; I don't know	Checkboxes	Reported			x	x
226. Congenital anomaly - surgery	Surgery already occurred or needed in the future due to the congenital anomaly	Y; N; I don't know	Radio buttons	Reported			x	x
227. Congenital anomaly - follow-up	Follow-up / evolution of the congenital anomaly in the first 8 weeks after birth	Text	Text box	Reported				x
**Neonatal withdrawal symptoms**								
228. Withdrawal symptoms	Identification of neonatal withdrawal symptoms, related to medication, drugs or alcohol use during pregnancy	Y; N; I don't know	Radio buttons	Reported			x	
229. Withdrawal symptoms - description	Description of the neonatal withdrawal symptoms, related to medication, drugs or alcohol use during pregnancy	Text	Notes box	Reported			x	
230. Withdrawal symptoms - duration	Duration of the neonatal withdrawal symptoms (or still ongoing)	Text	Notes box	Reported			x	

231. Withdrawal symptoms - medication	Medication used to alleviate the neonatal withdrawal symptoms	Y; N; I don't know	Radio buttons	Reported			x	
232. Withdrawal symptoms - type of medication	Names of the medicines used to alleviate the neonatal withdrawal symptoms	Text	Notes box	Reported			x	
**Neonatal department admission**								
233. Admission to a neonatal department	Admission to a neonatal department or a neonatal intensive care department	My baby was not admitted to a neonatal (intensive) unit after birth; My baby was admitted to a neonatal ward after birth (N); My baby was admitted to a neonatal intensive care unit (NIC) after birth	Radio buttons	Reported			x	
234. Neonatal department admission – reason	Reason of the admission to a neonatal department or a neonatal intensive care department	Preterm birth (< 37 weeks); Low birth weight (< 2500 grams); Oxygen deficiency at birth; Problems with breathing (specify); (congenital) problem with the metabolism (i.e., abnormal Guthrie test); Jaundice (icterus) for which phototherapy was needed; Problems with the heart (specify); An abnormally fast heart rhythm; An abnormally slow or irregular heart rhythm; Infection (specify); Problems with digestion (specify); Seizures or epileptic seizures; (severe) congenital abnormality; Stroke; Neonatal withdrawal symptoms due to the use of medication or substances during pregnancy; Problems with the temperature control; Hypotonia ; Other (specify) - Including possibility to add free text	Checkboxes with embedded text fields, Multiple choice - Notes box	Reported			x	
235. Neonatal department - current stay	Currently staying at a neonatal department	Y/N	Radio buttons	Reported			x	
236. Neonatal department - days	Number of days that the neonate (has) stayed at a neonatal department	Number	Text box	Reported			x	
**Feeding of the infant**								
237. Type of feeding	Type of feeding in the first 24 hours after birth (i.e., in the first postpartum questionnaire) or currently (i.e., in the second postpartum questionnaire)	Breastfeeding/breast milk; Formula feeding; Mixed feeding (breastfeeding/breastmilk and formula); Other (specify)	Radio buttons	Reported			x	x

238. Breastfeeding/giving breast milk – since birth	Breastfeeding or giving breast milk, ever since birth	Y/N	Radio buttons	Reported			x	
239. Breastfeeding/giving breast milk - currently	Currently breastfeeding or giving breast milk	Y/N	Radio buttons	Reported			x	
240. Breastfeeding/giving breast milk – exclusively	Exclusively breastfeeding or giving breast milk	Y/N	Radio buttons	Reported			x	
241. Breastfeeding/giving breast milk - duration	Duration of breastfeeding or giving breast milk	Text	Text box	Reported			x	
242. Breastfeeding/giving breast milk - reason for cessation	The reason of breastfeeding cessation or stop giving breast milk to the infant	Text	Notes box	Reported			x	
**Neonatal complications**								
243. Neonatal complications	Complications or medical problems that occurred in the infant in the first 28 days after birth	Problems with breathing (specify); (congenital) problem with the metabolism (i.e., abnormal Guthrie test); Jaundice (icterus) for which phototherapy was needed; Problems with the heart (specify); An abnormally fast heart rhythm; An abnormally slow or irregular heart rhythm; Infection (specify); Problems with digestion (specify); Seizures or epileptic seizures; (severe) congenital abnormality; Cerebral haemorrhage; Neonatal withdrawal symptoms due to the use of medication or substances during pregnancy; Problems with the temperature control; Hypotonia; Other (specify)	Checkboxes with embedded text fields, Multiple choice - Notes box	Reported			x	x
244. Neonatal hospital (re)admission	Hospital (re)admission of the infant in the first 28 days after birth (i.e., not the hospital admission for the birth itself)	Y/N	Radio buttons	Reported			x	x
245. Neonatal hospital (re)admission - reason	Reason of the hospital (re)admission of the infant in the first 28 days after birth	Text	Notes box	Reported			x	x
**246. Medication use of the neonate**	Names of the medicines used by the infant in the first 28 days after birth. If the name(s) is (are) not known, the indication can also be given.	Text	Notes box	Reported			x	x

Abbreviations : EM = Pregnancy Enrolment Questionnaire; FU = Pregnancy Follow-up Questionnaire; PP 1 = First Postpartum Questionnaire; PP 2 = Second Postpartum Questionnaire.

Notes: [1]Multiplication of the neonatal outcome fields in case of plurality; all the variables of category 7 need to be competed for each live born child; [2]Structured text field with autocomplete function, linked to NATO database with country names and alpha-2 codes.

2 Supplementary Figure

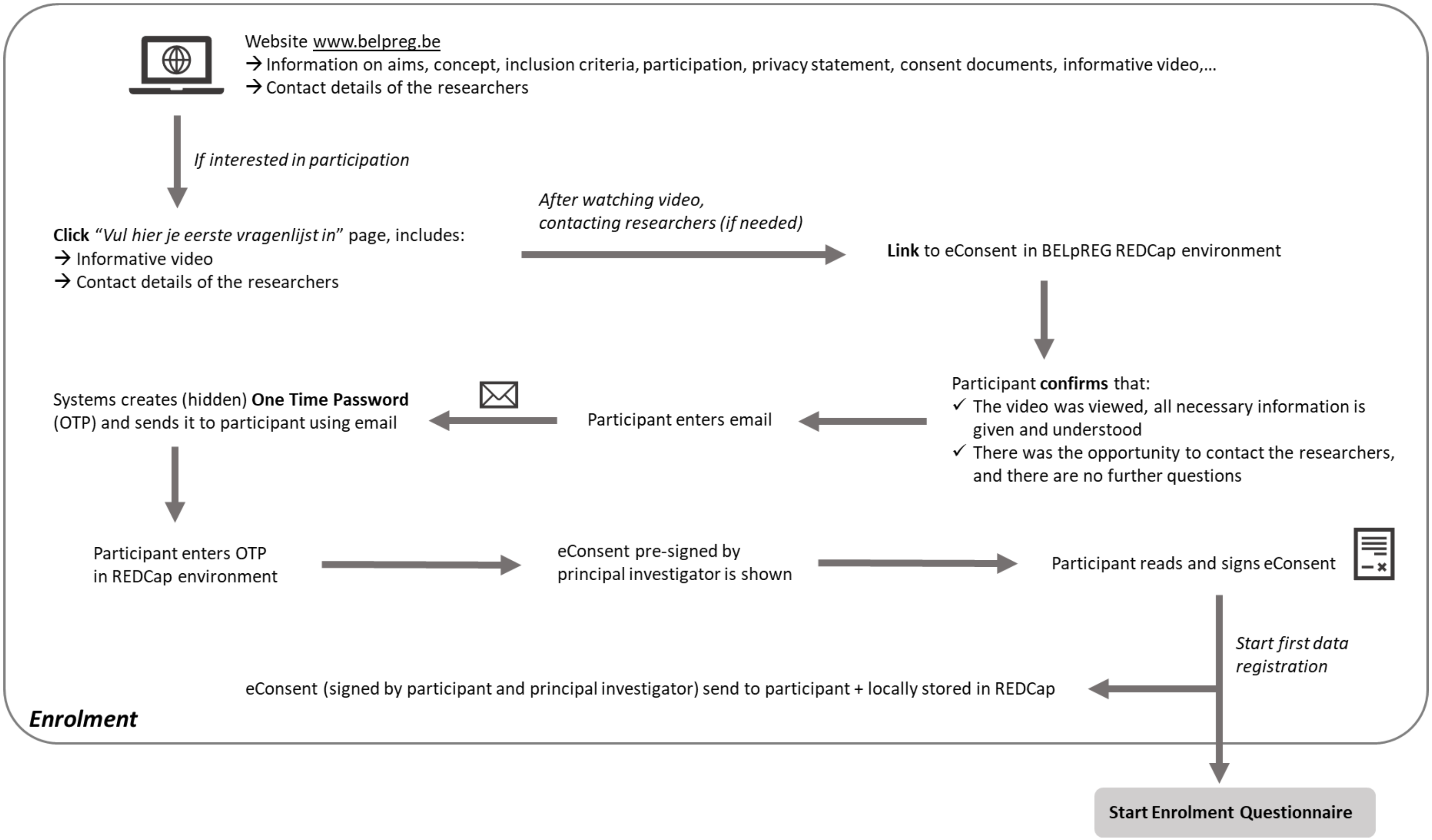

Supplementary Figure 1. Schematic overview of the eConsent procedure
